# Low‐grade oncocytoid neoplasm in the Milan system: Tips for the diagnosis of Warthin tumor and other differential diagnostic considerations

**DOI:** 10.1002/cncy.70037

**Published:** 2025-09-11

**Authors:** Esther Diana Rossi, Christopher C. Griffith

**Affiliations:** ^1^ Division of Anatomic Pathology and Histology Fondazione Policlinico Universitario “Agostino Gemelli”‐IRCCS Rome Italy; ^2^ Associate Professor of Pathology Cleveland Clinic Lerner College of Medicine Cleveland Ohio USA

**Keywords:** fine‐needle aspiration cytology, immunocytochemistry, oncocytic lesions, personalized medicine, salivary neoplasms

## Abstract

The current review article deals with the evaluation of the oncocytic/oncocytoid lesions in the salivary gland. The authors will focus on the diagnosis of Warthin tumor (WT) as a launching point to detail important morphologic findings that should prompt designation of an aspirate as oncocytic salivary gland neoplasm of uncertain malignant potential or other Milan categories. Oncocytic cells are defined as cells with a moderate to abundant amount of eosinophilic finely granular cytoplasm, round‐to‐oval nuclei, and large‐distinct nucleoli. In contrast, the term oncocytoid is also frequently used in this discussion and indicates tumor cells with similarly abundant and sometimes granular cytoplasm but lacking all the definitive features of a true oncocyte. Several helpful tips are provided in hopes of improving an accurate diagnosis of WT on an aspirate sample. Using these types allows for consideration of important differential diagnoses, including both benign and malignant entities, when faced with an oncocytic salivary gland neoplasm. The morphological criteria as well as the possible application of ancillary techniques are also discussed.

## Abstract 2

This review examines the evaluation of oncocytic/oncocytoid lesions in salivary glands and focuses on the diagnosis of Warthin tumors to detail important morphological findings that should prompt the designation of an aspirate as an oncocytic salivary gland neoplasm of uncertain malignant potential or another Milan category. Several helpful tips are provided to improve accuracy in the diagnosis of Warthin tumors from aspirate samples. Morphological criteria and the possible application of ancillary techniques are also discussed.

## INTRODUCTION

The fifth edition of the World Health Organization (WHO) classification of head and neck tumors recognizes more than 15 benign and 20 malignant neoplasms.^1^, ^2^, ^3^ Salivary gland lesions encompass a wide spectrum of morphologically heterogeneous neoplasms, which can pose significant diagnostic challenges on both histologic and cytologic evaluation. The primary role of fine‐needle aspiration (FNA) cytology is to guide appropriate management with specific diagnosis a secondary goal.^1^, ^2^ The introduction of the Milan System for Reporting Salivary Gland Cytopathology (MSRSGC) offers a standardized classification scheme placing aspirates into diagnostic categories with defined risk of malignancy (ROM) and clinical/surgical management recommendations.^4^, ^5^, ^6^

In the current review, we will discuss only the possible differential diagnosis in samples with prevalence of oncocytic/oncocytoid features. We will focus on the diagnosis of Warthin tumor (WT) as a launching point to detail important morphologic findings that should prompt designation of an aspirate as oncocytic salivary gland neoplasm of uncertain malignant potential (SUMP) or other Milan categories. In our discussion, oncocytic cells are defined as cells with at least a moderate amount of eosinophilic and finely granular cytoplasm, round‐to‐oval nuclei and large‐distinct nucleoli. The term oncocytoid is also frequently used to indicate tumor cells with some oncocytic features including abundant and sometimes granular cytoplasm but lacking all the definitive features of a true oncocyte.

### MSRSGC

The nomenclature of the six diagnostic categories in the MSRSGC remains unchanged in the second edition, published July 2023.^7^, ^8^ The six categories include: Non‐Diagnostic, Non‐Neoplastic, Atypia of Undetermined Significance (AUS), Neoplasm, Suspicious for Malignancy (SFM), and Malignant. Importantly, the Neoplasm category is subdivided into Benign Neoplasm and SUMP. The ROM associated with each of these categories has been refined based on a growing volume of Milan‐specific literature.^4^, ^5^, ^9^, ^10^, ^11^, ^12^, ^13^, ^14^, ^15^ There have also been efforts to further subclassify indeterminate aspirates including AUS, SUMP, and SFM. Briefly, the AUS category is mostly adopted for those problematic salivary gland FNA samples encountered in routine practice that lack qualitative and/or quantitative cytomorphologic features required for the definitive diagnosis of a neoplasm, yet contain atypical cytologic features that precludes being classified as “Non‐Neoplastic.”^7^, ^8^, ^16^, ^17^, ^18^ For example, this category may include oncocytic cells that could represent oncocytic hyperplasia/oncocytosis or a bland oncocytic neoplasm. Among the criteria for a diagnosis of AUS, the Milan System also includes limited samples with scant oncocytic or oncocytoid cells.^7^, ^8^ This AUS diagnosis includes a variety of nonneoplastic and neoplastic lesions that cannot be further classified based on qualitative and quantitative limitations.^7^, ^8^ Salivary gland aspirates with metaplastic changes including squamous, oncocytic, mucinous, and sebaceous can be challenging and raise the differential diagnosis of a poorly sampled neoplasm including MEC, pleomorphic adenoma (PA), or WT. The ROM for the AUS category in the second edition of the Milan System is approximately 30%, with some variability depending on the subgroup of AUS.^4^ From a clinical management standpoint, salivary aspirates classified as AUS due to scant oncocytic cells may be best managed with repeat sampling or clinical follow‐up depending on the clinical and radiologic concern for a more neoplastic process. Excision may be performed when a neoplasm continues to be suspected clinically.

Within the Neoplasm categories, the Benign Neoplasm subgroup should be reserved for cases in which a specific benign entity can be definitively diagnosed. Among oncocytic lesions (Table 1), WT represents the most frequently encountered benign neoplasm.

**TABLE 1:** Oncocytic lesions and ICC expression.

	Positive ICC	Negative ICC
WT	P63 P40	S100 Mammaglobulin DOG1 GATA‐3 SOX10 PanTrak NR4A3 Androgen receptor PLAG1 HMGA2
Oncocytosis/oncocytoma	P63 P40	S100 Mammaglobulin DOG1 GATA‐3 SOX10 PanTrak NR4A3 Androgen receptor PLAG1 HMGA2
Acinic cell Ca	DOG1, NR4A3 SOX10	S100 Mammaglobulin GATA‐3 PanTrak P63 P40 Androgen receptor PLAG1 HMGA2
Secretory Ca	SOX10 S100 PanTrak Mammaglobulin	DOG1 NR4A3 GATA‐3 P63 P40 Androgen receptor PLAG1 HMGA2
Oncocytic MEC	P63 P40	S100 Mammaglobulin DOG1 +/–GATA‐3 SOX10 +/–PanTrak NR4A3 Androgen receptor PLAG1 HMGA2
Salivary duct Ca	Androgen receptor GATA‐3	S100 Mammaglobulin +/–DOG1 SOX10 PanTrak NR4A3 PLAG1 +/–HMGA2 +/–P63 P40

*Note:* Adapted from the Milan System Edition II.^8^

Abbreviations: Ca, carcinoma; ICC, immunocytochemistry; MEC, mucoepidermoid carcinoma; WT, Warthin tumor.

The SUMP subcategory is used for aspirates diagnostic of a neoplasm but for which a definitive diagnosis is not possible, and the differential includes both benign and malignant tumors.^7^, ^8^, ^19^, ^20^, ^21^ SUMP can be subcategorized to include cellular basaloid neoplasms, cellular oncocytic/oncocytoid neoplasms, cellular neoplasms with clear cell features, and cellular neoplasms with mixed features.^19^, ^20^, ^21^ Classification of an aspirate as SUMP leads to more complex clinical decision‐making. Like the Benign Neoplasm category, correlation with preoperative imaging should be performed to define the detailed size and extent of the tumor and address the possibility or regional metastases to cervical nodes. Because the SUMP category has a low ROM (approximately 35%), conservative surgical excision is typically recommended.^8^ Intraoperative evaluations can also be helpful in many cases to further classify the tumor, guide extent of excision, evaluate margin status, and determine potential need for a neck dissection.

### Warthin tumor

**FIGURE 1:**
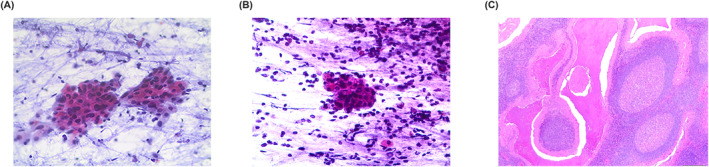
(A and B) The cytological features of a Warthin tumor (Pap stain, 40×). The cells are organized in clusters of oncocytes with a dirty background including small lymphocytes. (C) The histological features of dense lymphoid stromal component and layers/bilayers of oncocytic cells (H & E, 40×). Pap indicates Papanicolaou.

WT is the second most common salivary neoplasm and is exclusively found in the parotid gland/peri‐parotid lymph nodes.^7^, ^8^ Most patients are in their sixth to seventh decade and almost all of them have a long smoking history. The clinical appearance is of a doughy, cystic, painless mass, fluctuating in size, and frequently bilateral. The cytological diagnosis of WT is based on the recognition of three essential parameters including: 1) dirty proteinaceous background, 2) prevalence of small lymphocytes, and 3) clusters and sheets of uniform oncocytes. The oncocytes show typical features of abundant granular and eosinophilic cytoplasm, well‐defined cell borders, and medium‐sized nuclei with prominent nucleoli (Figure 1A–C).

Occasionally, WTs may show infarction, which is followed by a rapid increase in size, clinically raising the possibility of a more aggressive salivary gland malignancy. Infarction can further complicate the cytologic and/or histologic evaluation due to the presence of necrotic debris and atypical appearing reactive squamous metaplasia.^7^, ^8^, ^22^ WT may contain necrotic debris and atypical squamous cells. The latter give raise to possible misinterpretation as squamous cell carcinoma (SCC), which is composed of a larger number of atypical squamous cells with higher mitotic activity than is characteristically seen in WT.^23^, ^24^, ^25^, ^26^, ^27^

In general, the cytologic diagnoses of Benign Neoplasm, especially that of WT, should be combined with the clinical and radiologic findings. It is also important to emphasize that asymptomatic patients with WT may be managed conservatively with clinical follow‐up, given the tumor’s negligible risk of malignant transformation. The correct diagnosis of WT is therefore crucial as a misdiagnosis of a low‐grade carcinoma as WT could result in nonsurgical treatment and potential progression of disease.^25^, ^26^, ^27^

Data from the literature have demonstrated that the malignant transformation of WT is exceptionally rare with a reported incidence of approximately 0.3%.^25^, ^26^, ^27^ It is more commonly associated with a transformation of the lymphoid component to a malignant lymphoma than an epithelial malignant transformation.^24^ The criteria for such malignant transformation include 1) a preexisting WT; 2) evidence of a transition from benign oncocytic to malignant epithelium; 3) evidence of infiltrative growth into surrounding lymphoid tissue; and 4) absent clinical history of an extra‐salivary primary carcinoma.^23^, ^24^, ^25^, ^26^, ^27^

#### WT versus oncocytoma

**FIGURE 2:**
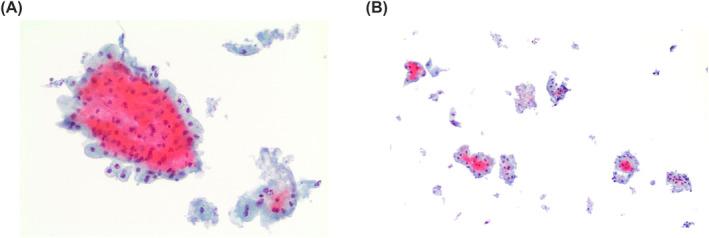
The figures show the details of clusters of oncocytic cells in oncocytoma. Note the different size and shapes of these clusters and the lack of inflammatory background and lymphocytes (A) Pap stain, 20×. (B) Pap stain, 40×. Pap indicates Papanicolaou.

Oncocytoma and WT are both benign oncocytic neoplasms and can show overlapping cytologic features.^7^, ^8^, ^28^, ^29^, ^30^, ^31^ Cytologic criteria for oncocytoma include 3D‐clusters and sheet of oncocytic cells, with well‐defined cytoplasmic borders, prominent nucleoli, and a clean background without lymphocytes and cystic components (Figure 2A,B). Although the cellular features are essentially identical between oncocytoma and WT, the differentiating feature is the lack of a dirty proteinaceous background and prominent lymphoid population seen in WT. On the other hand, oncocytoma is morphologically indistinguishable from nodular oncocytosis/nodular oncocytic hyperplasia.^30^ Radiologic correlation is helpful as oncocytoma typically shows a circumscribed mass whereas nodular oncocytosis is characterized by multiple oncocytic nodules ranging in size from 0.2 to 2.5 cm in one or both parotid glands.

#### WT versus other oncocytic/oncocytoid neoplasms

**FIGURE 3:**
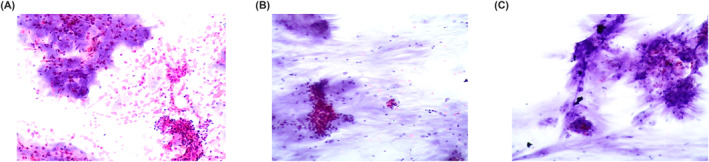
Figures show the morphological features of typical pleomorphic adenoma. The criteria include abundant fibrillary‐chondromyxoid stromal component embedded with myoepithelial cells, and at the periphery, small clusters of basaloid ductal cells with round nuclei and scant cytoplasm (Pap stain, 40×). Pap indicates Papanicolaou.

**FIGURE 4:**
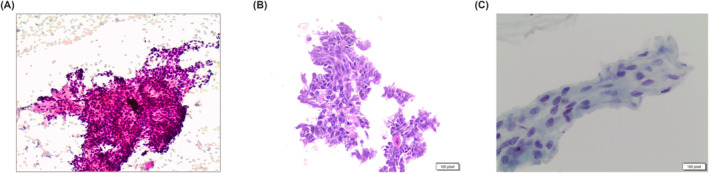
(A and B) The details of myoepithelioma with moderate atypia and different shapes as appreciated in the pictures. Medium sized nuclei with some nuclear clearing and poorly defined cell borders (Pap stain, 40×). (C) The same features on a cell‐block preparation from the same case (H & E, 40×). Pap indicates Papanicolaou.

Although WT and oncocytoma are the most common entities showing exclusively, or almost exclusively, oncocytic cells, an oncocytic/oncocytoid pattern can be seen in a variety of other neoplasm including PA (Figure 3A–C), myoepithelioma (Figure 4A–C), and MEC.^7^, ^8^, ^28^, ^29^, ^30^, ^31^, ^32^, ^33^ It is also important to remember that some nononcocytic neoplasms, such as acinic cell carcinoma (AciCC), secretory carcinoma, and metastatic renal cell carcinoma can be morphologically misinterpreted as oncocytic (i.e., oncocytoid).^7^, ^8^, ^28^, ^29^, ^30^, ^31^, ^32^, ^33^ Even though most of these cases have specific morphological features allowing accurate recognition, when a neoplasm cannot be further subclassified, the SUMP category is strongly suggested and a differential diagnosis including predicted tumor grade should be given.

Oncocytic MEC, Warthin‐like MEC, and standard MEC can frequently show overlapping cytologic features with WT.^7^, ^8^, ^23^, ^24^, ^25^, ^26^, ^27^, ^28^, ^29^, ^30^, ^31^, ^32^, ^33^ MEC are defined by the presence of epidermoid, mucinous, and intermediate cells. In many cases, the epidermoid cells in MEC can have an oncocytoid appearance but some examples can contain true oncocytes. When combined with the common finding of a lymphoid response, such aspirates are easily misinterpreted as WT.^7^, ^8^, ^27^, ^28^, ^29^, ^30^, ^31^, ^32^, ^33^ Clinical and radiologic findings can be useful to provide evidence of an infiltrative cancer but most cases of low‐ and intermediate‐grade MEC will have circumscribed radiographic appearance.^7^, ^8^, ^27^, ^28^, ^29^, ^30^, ^31^, ^32^, ^33^ Nuclear atypia, mitotic activity, and necrosis are rare findings in even high‐grade MEC but when present are suggestive of malignancy. Unfortunately, immunostains are not helpful in the distinction between WT and MEC because both show a mixture of p63 positive and negative tumor cells. In some clinical situations, when the preoperative distinction between WT and MEC is critical, molecular studies are potentially helpful. Most MEC have a t(11;19) translocation with *CRTC1*:*MAML2* gene fusion whereas a minority harbors a t(11;15) translocation with *CRTC3*:*MAML2*. These rearrangements are not identified in WT.^34^

**FIGURE 5:**
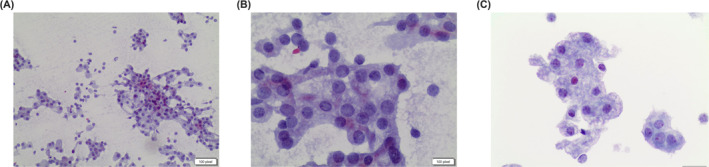
The pictures show details of an acinic cell carcinoma. The cells show mild atypia, oncocytoid, and granular cytoplasm. (Pap stain, 40×). Pap indicates Papanicolaou.

AciCC are usually composed of cytologically low‐grade tumor cells with abundant cytoplasm containing zymogen granules that can give a granular appearance like oncocytes (Figure 5A–C).^7^, ^8^, ^35^, ^36^, ^37^ Additionally, AciCC show a prominent lymphocytic response in approximately 30% of cases further overlapping with WT. An accurate evaluation of the morphological features of AciCC can be achieved by recognizing some of the key cytologic features including coarsely granular cytoplasm with indistinct cytoplasmic borders, enlarged nuclei, and loosely cohesive cells. AciCC also have fragile cytoplasm resulting in the common finding of naked nuclei and granular background debris. In challenging cases, immunostains can be helpful in this distinction between AciCC and WT with AciCC being positive for SOX10, DOG‐1, and NR4A3.^37^, ^38^, ^39^, ^40^

Concerning NR4A3, the NR4A3 translocation is present in the majority of AciCC, and NR4A3 immunomarker is an effective surrogate marker for the translocation.^39^ Published literature shows that nuclear expression of NR4A3 has high sensitivity and specificity for AciCC in cytologic material, performing better than both DOG‐1 IHC and NR4A3 fluorescence in situ hybridization.^39^ Because NR4A3 is not expressed in normal acinar cells, it can also be used to distinguish AciCC from normal acinar cells and to support a diagnosis of AciCC in poorly differentiated/dedifferentiated cases.^39^

**FIGURE 6:**
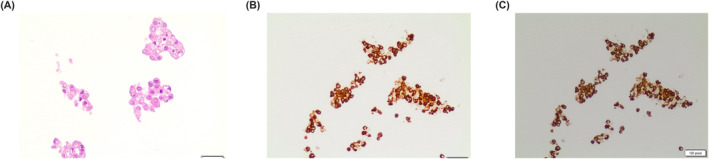
(A) The cytological details of a secretory carcinoma. The cells are defined by an eosinophilic‐vacuolized cytoplasm, with roundish vacuoles. Bland nuclear features (Papanicolaou stain, 40×). (B) Positivity for mammaglobin (cell block section, 40×). (C) Positivity for S100 (cell block section, 40×).

Secretory carcinoma is another low‐grade salivary gland carcinoma that potentially mimics WT on aspirate material.^7^, ^8^, ^41^, ^42^ Secretory carcinoma typically shows cellular smears with single cells as well as follicular and papillary architecture (Figure 6A). The cells show single or multiple cytoplasmic vacuoles but can also have the appearance of granular cytoplasm as seen in oncocytes. Immunohistochemical positivity for S100 and mammaglobin (Figure 6B) as well as MUC4 can be helpful in confirming the diagnosis of secretory carcinoma.^43^, ^44^ Most secretory carcinomas show an *ETV6::NTRK3* gene fusion, and therefore molecular or an immunostain for pan‐TRK can be helpful also.^43^, ^44^

**FIGURE 7:**
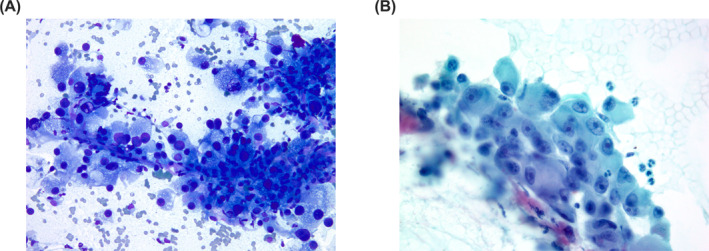
Figures show morphologic features of a metastatic renal cell carcinoma to the parotid gland. It is composed of solid clusters of cells with vacuolized cytoplasm, clear cytoplasmic features with an oncocytic appearance (Diff‐quick and Pap stain, 40×). Pap indicates Papanicolaou.

Metastases to the parotid gland from nonhead and nonneck sites are uncommon but when it does occur, can show features suggestive of an oncocytic neoplasm.^45^, ^46^, ^47^ In particular, the morphological features of a metastatic renal cell carcinoma comprise moderate to high cellularity, characterized by single cells and small clusters, fragmented papillae, or sheets (Figure 7A,B). The cells show abundant pale, finely granular, clear or vacuolated cytoplasm, with round‐to‐oval nuclei with large nucleoli. CD10, PAX8, and anti‐RCC reactivity is useful for the diagnosis. Furthermore, the correlation with the clinical and radiological history is crucial for the correct diagnosis.

### High‐grade tumors can also show oncocytic features

**FIGURE 8:**
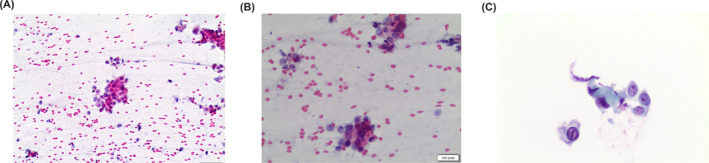
Figures show the details of a high‐grade carcinoma with oncocytoid features from a case of salivary duct carcinoma. Large pleomorphic nuclei, consistent nucleoli, scant to moderate eosinophilic cytoplasm. (Pap stain and liquid‐based cytology, 40×). Pap indicates Papanicolaou.

Oncocytic/oncocytoid features are frequently encountered in malignant primary salivary gland and metastatic tumors.^7^, ^8^, ^45^, ^46^, ^47^ Salivary duct carcinoma (SDC) is an uncommon tumor but represents the most common high‐grade primary salivary gland carcinoma.^7^, ^8^, ^48^, ^49^, ^50^ Aspirates of SDC should be recognized as a high‐grade malignancy and is characterized by cells with large, pleomorphic nuclei containing prominent nucleoli and having abundant oncocytoid cytoplasm with well‐defined borders (Figure 8A–C). Mitotic figures, including atypical forms and necrotic debris, are frequently evident. In the setting of a high‐grade adenocarcinoma of the salivary gland, identification of nuclear positivity for androgen receptor (AR) is helpful to confirm the diagnosis of SDC. However, one must be cautious in the use of AR, because reactivity can be seen in the setting of benign apocrine metaplasia seen in a variety of low‐grade salivary gland neoplasm as well.^7^, ^8^, ^48^, ^49^, ^50^

Metastases of poorly differentiated SCC (often of cutaneous origin) to the parotid can also give the appearance of a high‐grade oncocytoid neoplasm.^45^, ^46^, ^47^, ^51^ Immunostains can be helpful to confirm squamous phenotype, and a review of clinical history can confirm a history in many cases.

### Tips for the diagnosis of WT on aspirate samples

#### Tip 1: Mind the mucus

The presence of mucus on an aspirate of an oncocytic salivary gland neoplasm should prompt caution in making a definitive diagnosis of a benign WT. Although some mucus is acceptable in aspirates of WT, its presence should at least prompt consideration for the possibility of MEC or another adenocarcinoma. If there is a small amount of mucus and all the other cytologic features are classic for the diagnosis of WT, then this can be accepted as mucinous metaplasia allowing classification of the aspirate as a benign neoplasm in the Milan system. However, in our opinion, the presence of significant mucus or a lack of all other classical WT cytologic features should prompt classification of the aspirate as oncocytic SUMP with a differential diagnosis including WT as well as carcinoma (primarily MEC) as dictated by the overall findings. Mucus in salivary gland aspirates is of enough concern that its presence in an acellular aspirate should prompt a Milan classification of AUS rather than nondiagnostic.

#### Tip 2: Look out for lymphocytes

Lymphoid stroma is a key element in the diagnosis of WT. Unfortunately, in the setting of a low‐grade oncocytic neoplasm on aspiration, the presence of a prominent lymphoid background can prompt a cytopathologist to too quickly jump to a diagnosis of WT. We therefore recommend caution relying too heavily on the presence of a lymphoid background to make the diagnosis of WT. Although the presence of this lymphoid background, combined with “grungy” cystic debris, is a classic feature of WT, a prominent lymphoid is also common for several salivary gland carcinomas. Most importantly for this discussion, MEC often have a prominent tumor associated lymphoid response and can show a lymphoid background on aspirate material. When combined with the finding of clusters of low‐grade oncocytoid/epidermoid tumor cells, this can give the erroneous impression of a WT. Similarly, AciCC frequently may show a lymphoid component but the tumor cells in this entity are less likely to mimic oncocytes but can on occasion. One very helpful bit of clinical information is the patient’s age, because WT is extremely rare in patients under 40 years of age and in nonsmokers, whereas low‐grade salivary gland carcinomas can occur in younger patients.

#### Tip 3: Be cautious about cohesion

Aspirates of WT and oncocytoma are characterized by the presence of oncocytes in clusters. Cytologically, these tumor cells are typically bland but may demonstrate some reactive atypia and/or metaplastic changes. An important architectural feature of these clusters is also that they should be tightly cohesive and well organized, typically suggesting a flat sheet of cells. In contrast, aspirates of low grade oncocytoid carcinomas such as MEC will usually show a loss of cohesion with less tightly packed cells and occasional single cells falling off the cell clusters. In cases of AciCC, this loss of cohesion is also associated with more fragile cytoplasm resulting in naked nuclei and a granular background of cytoplasmic debris.

## CONCLUSION

The presence of oncocytic/oncocytoid cells is frequently encountered in aspirates of salivary gland lesions. The diagnosis of WT can be made accurately on aspirate material with classic features to allow appropriate clinical management. However, there are a variety of low‐grade salivary gland carcinomas that can closely mimic WT on cytology. It is therefore imperative to be aware of the overlapping cytomorphologic features that can lead to diagnostic pitfalls. We present three helpful tips for the evaluation of these aspirates in the hope of helping readers avoid these errors. For example, the presence of lymphocytes, a key cytologic feature of WT, can be seen in other entities and therefore this feature, if used alone, is insufficient for a diagnosis of WT. Prominent mucus and lower cellular cohesion are often clues to an alternative diagnosis. Although some WT can show atypical features, it is best to classify an aspirate as SUMP if the cytologic features raise the possibility of another entity.

## CONFLICT OF INTEREST STATEMENT

The authors declare no conflicts of interest.
